# Return to Activities of Daily Living after Breast Cancer Surgery: An Observational Prospective Questionnaire-Based Study of Patients Undergoing Mastectomy with or without Immediate Reconstruction

**DOI:** 10.1155/2023/9345780

**Published:** 2023-09-20

**Authors:** L. Ballance, R. L. Wilson, C. C. Kirwan, G. Boundouki, V. P. Taxiarchi, B. G. Baker, V. Rusius, M. Rowland, J. R. Henderson, N. Marikakis, J. McAleer, J. R. Harvey, On Behalf of the Northwest Breast Research Collaborative

**Affiliations:** ^1^The Nightingale Breast Centre, Wythenshawe Hospital, Manchester University NHS Foundation Trust, Southmoor Road, Manchester M23 9LT, UK; ^2^Division of Cancer Sciences, School of Medical Sciences, Faculty of Biology, Medicine and Health, University of Manchester, Manchester Academic Health Science Centre, Manchester, UK; ^3^Sheffield Breast Unit, Royal Hallamshire Hospital, Sheffield S10 2JF, UK; ^4^Centre for Women's Mental Health, Division of Psychology and Mental Health, Faculty of Biology, Medicine and Health, University of Manchester, Manchester, UK; ^5^Burnley Breast Unit, Burnley General Hospital, East Lancashire Hospitals NHS Trust, Casterton Avenue, Burnley BB10 2PQ, UK; ^6^Liverpool Breast Unit, Linda McCartney Centre, Royal Liverpool University Hospital, Prescot Street, Liverpool L7 8XP, UK; ^7^Queen Alexandra Hospital, Portsmouth Hospitals NHS Trust, Southwick Hill Road, Cosham, Portsmouth, Hampshire PO6 3LY, UK; ^8^Breast Care Centre, Ainscoe House, Blackpool Victoria Hospital, 12 E Park Dr, Blackpool FY3 8DX, UK

## Abstract

**Background:**

Patients often ask about the time taken to return to activities of daily living (ADLs) after breast surgery, but there is a lack of data to give accurate guidance. We aimed to assess the feasibility of a study to determine the time taken to return to ADLs after mastectomy with or without breast reconstruction.

**Materials and Methods:**

A prospective multicentre, self-reported questionnaire-based feasibility study of women who had undergone mastectomy ± reconstruction was performed, between Jan 2017 and Dec 2019. Women were asked to self-report when they returned to 15 ADLs with a 5-option time scale for “return to activity.”

**Results:**

The questionnaire was returned by 42 patients (median [range] age: 64 [31–84]). Of these, 22 had simple mastectomy, seven mastectomy and implant reconstruction, seven mastectomy and autologous reconstruction (DIEP), and six did not specify. Overall, over 90% could manage stairs and brush hair by two weeks and 84% could get in and out of the bath by four weeks. By 1-2 months, 92% could do their own shopping and 86% could drive. 68% of women employed returned to work within four months. Compared to simple mastectomy, patients undergoing reconstruction took a longer time to return to getting in/out of bath (<2 vs. 2–4 weeks), vacuuming (2–4 weeks vs. 1-2 months), and fitness (1-2 vs. 3-4 months). There was a slower return to shopping (1-2 months vs. 2–4 weeks), driving and work (both 3-4 vs. 1-2 months), and sports (3-4 vs. 1-2 months) in autologous reconstruction compared to implant reconstruction.

**Conclusion:**

This study is feasible. It highlights slower return to specific activities (particularly strength-based) in reconstruction patients, slower in autologous compared with implant reconstruction. The impact on return to ADLs should be discussed as part of the preoperative counselling as it will inform patients and help guide their decision making. A larger study is required to confirm these results.

## 1. Introduction

Over 55,000 women are diagnosed with breast cancer per year in the UK, the majority of whom undergo surgery [1]. The 2008 UK national mastectomy and breast reconstruction audit estimated only 21% of women were undergoing immediate reconstruction; in the following years, this increased by 50% and approximately 85% of these reconstructions are implant based [2, 3]. Informed consent requires delivery of information on the postoperative impact of surgery and the length of time taken to return to activities of their daily living. These are the activities of daily basic hygiene, performing household tasks, driving, exercising, and returning to work. The literature contains few data on the length of time taken for return to these activities after breast surgery. Most studies focus on return to work (RTW) data or quality of life (QoL) data [4–8]. Rates of return to work in a prospective study of women diagnosed with breast cancer in South America were 30.4% and 60.4% at 12 and 24 months [4]. Women who underwent breast conserving surgery had higher return to work rates than those that underwent mastectomy, regardless of reconstruction procedures [4]. Other studies focus on recovery for all breast cancer survivors, not surgical patients specifically [5]. No data are available on functional activities such as managing hygiene, mobilising, and socialising.

There is no consensus on when patients should be returning to activities of daily living after breast surgery. A qualitative study exploring the perceptions of women treated for breast cancer found returning to normal activities was longer than either the women's or their physicians' expectations [7]. Also the women in this study felt the physical impact the surgery had on activities led to a psychological strain as they were constantly reminded of their illness [7]. After breast reduction surgery, the advice on returning to work and driving varies amongst health professionals, with 33% of plastic surgeons giving no information on returning to driving and 12% giving no advice on returning to work [5, 7].

Approximately 50% of all breast cancers are diagnosed in women under the age of 65 and 20% under the age of 50, and they are therefore more likely to be in employment or have younger children [1]. There is a need to accurately set expectations for recovery from breast surgery. This will aid patients in making decisions especially surrounding reconstruction. We aimed to determine the feasibility of running a study to establish time taken after mastectomy, with or without reconstruction, to return to key ADLs and identify any patient-reported factors that may affect this.

## 2. Materials and Methods

The questionnaire was developed with a patient-led focus group of seven patients who had previously undergone mastectomy ± reconstruction. Through discussions, themes were developed, and 15 key activities of daily living were identified as being important to these women, Table 1. A questionnaire for the feasibility study was designed with the focus group. The questionnaire contained basic demographic questions, questions on five ADL themes (personal hygiene, transportation and shopping, managing household, fitness, and work) split into 15 activities and a free text question about any factors which may have delayed their return to ADLs. This is a nonvalidated questionnaire. A time scaled table (<2 weeks, 2–4 weeks, 1-2 months, 3-4 months, and >4 months or not attempted) was used to indicate at what time point they had recovered sufficiently to complete the activity (Supplementary Table 1). The questionnaire was trialled in two breast units, with written and verbal feedback from patients. Further amendments and review from a patient involvement group, research active surgeons, and statisticians were undertaken before being used in the study.

Patients undergoing a mastectomy with or without breast reconstruction between 2017 and 2019 were recruited from six hospitals. Patients were approached prior to surgery and asked to prospectively complete the questionnaire which documented age, operation, and employment type (manual, desk, mixed, and retired). Patients were then asked to complete the scale on return to 15 different ADLs: get in the bath unassisted, get out of bath unassisted, brush your hair, lift a full kettle, climb the stairs unassisted, pick up a child from the floor, vacuum the house, gardening, socialise outside of the house, do your own shopping, drive, return to work, perform the exercises comfortably, wear usual bras, and go back to the gym/playing sports. An open style question was used for patient comments on delay to return to these activities. They returned the questionnaire via a prepaid envelope to the study team, after four months or sooner if it was already complete. The study was approved by the NHS research ethics committee (17/NI/0158).

### 2.1. Statistical Analysis

Categorical variables are presented by frequencies and percentages, and information on age is given by the mean, range, and standard deviation. Stacked bar graphs and scatter plots were produced to display time to return to ADLs overall and by surgical operation type. Analysis was developed following exclusion of participants whose operation method was not recorded.

## 3. Results

### 3.1. Demographics

The questionnaire was completed by 42 women across six hospitals. The mean age was 64 years (range 31–84, SD 12.2), with 22 (52%) undergoing simple mastectomy, 14 (33%) undergoing reconstruction (seven implant based and seven autologous (DIEP)), and six not recording their operation in the questionnaire.

### 3.2. Return to ADLs in All Patients Undergoing Mastectomy (with or without Reconstruction, Implant, or Autologous Reconstruction)

**Figure 1:**
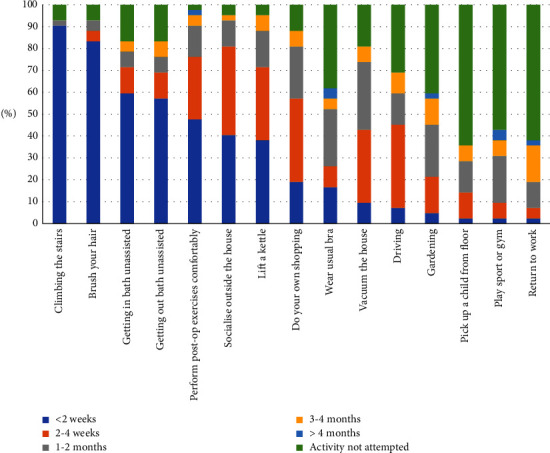
Time taken to return to activities of daily living after breast surgery. Data are presented as the percentage of women who were able to perform the ADL at each time point including those who did not attempt the activity during the follow-up period.

Overall, of those attempting the activity, by two weeks the majority of the participants could climb the stairs (*n* = 38, 97%) and brush their hair (*n* = 35, 90%). By four weeks, most participants could get in (*n* = 30, 86%) and out of the bath (*n* = 29, 82%). By two months, most participants could lift a kettle (*n* = 37, 93%), pick up a child (*n* = 12, 80%), vacuum (*n* = 31, 91%), socialise outside of the house (*n* = 39, 97%), do their own shopping (*n* = 34, 92%), and drive (*n* = 25, 86%). By four months, the majority had started gardening (*n* = 24, 96%), playing sports (*n* = 16, 89%), and returned to work (*n* = 15, 94%) (Figure 1, Supplementary Table 2).

### 3.3. Personal Hygiene

**Figure 2:**
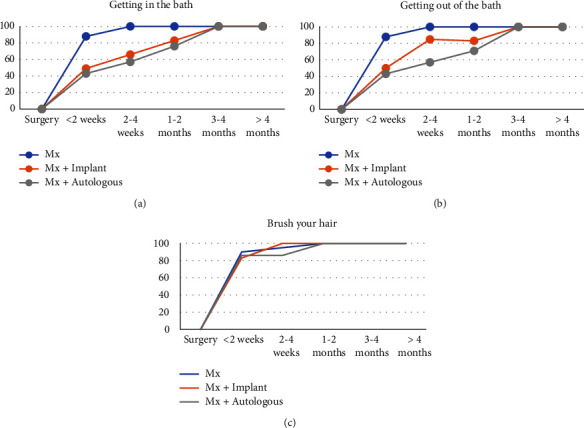
Time taken to return to activities of personal hygiene: (a) getting in the bath (Mx = 16, Mx + implant = 6, and Mx + autologous = 7), (b) getting out of the bath (Mx = 16, Mx + implant = 6, and Mx + autologous = 7), and (c) brushing your hair (Mx = 20, Mx + implant = 6, and Mx + autologous = 7), comparing simple mastectomy, implant-based reconstruction, and autologous reconstruction. Data are presented as the percentage of women who are able to perform the ADL at each time point. The number in brackets is the number of women who attempted this ADL during the recovery period.

All patients (*n* = 16, 6 did not attempt the activities) who underwent a simple mastectomy were able to attend to their personal hygiene by four weeks compared to 66% (*n* = 4, 1 did not attempt the activities) in the implant reconstruction group and 57% (*n* = 4) in the autologous reconstruction group (Figure 2). The majority, 88%, of patients who underwent a simple mastectomy could get in (*n* = 14) and out of a bath (*n* = 14) by two weeks, compared to 50% (*n* = 3) in the implant reconstruction group and 43% (*n* = 3) in the autologous group. The implant reconstruction group took up to 1-2 months to do these two activities and the autologous group 3-4 months compared to 2–4 weeks in the simple mastectomy group. All patients who underwent an implant reconstruction could brush their hair by 2–4 weeks (*n* = 6, one did not attempt this activity) compared to 86% in the autologous reconstruction group (*n* = 6).

### 3.4. Transportation and Shopping

**Figure 3:**
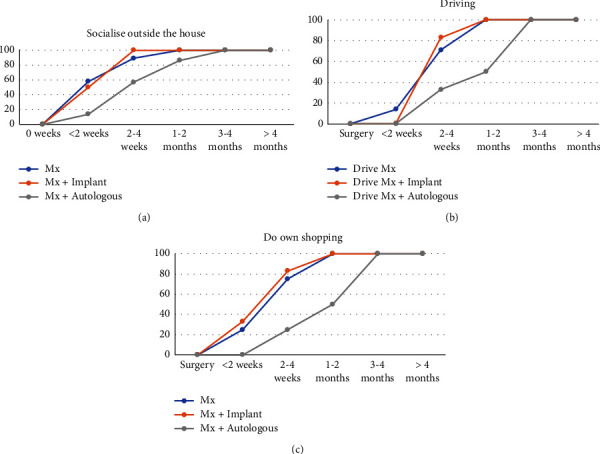
Time taken to return to activities of transportation and shopping: (a) socialising outside of the house (Mx = 21, Mx + implant = 6, and Mx + autologous = 7), (b) driving (Mx = 14, Mx + implant = 6, and Mx + autologous = 6), and (c) doing own shopping (Mx = 18, Mx + implant = 6, and Mx + autologous = 7), comparing simple mastectomy, implant-based reconstruction, and autologous reconstruction. Data are presented as the percentage of women who are able to perform the ADL at each time point. The number in brackets is the number of women who attempted this ADL during the recovery period.

Patients who underwent mastectomy or mastectomy and implant reconstruction were able to return to activities of transportation and shopping quicker than those who underwent autologous reconstruction (Figure 3). Of the patients who underwent a simple mastectomy, 90% (*n* = 19, 3 did not attempt this activity) could socialise outside of the house by 4 weeks compared to 100% (*n* = 6, one did not attempt this activity) who underwent implant reconstruction and 57% (*n* = 4) who underwent autologous reconstruction. In the mastectomy group and implant reconstruction group, all patients were able to complete their own shopping (*n* = 18, 4 did not attempt this activity, and *n* = 6, one did not attempt this activity, respectively) and drive (*n* = 14, 8 did not attempt this activity, and *n* = 6, one did not attempt this activity, respectively) by 1-2 months compared to 57% (*n* = 4) and 50% (*n* = 3, one did not attempt this activity), respectively, in the autologous group. All patients that underwent autologous reconstruction could socialise outside the house, complete their own shopping, and drive by 3-4 months.

### 3.5. Managing Household

**Figure 4:**
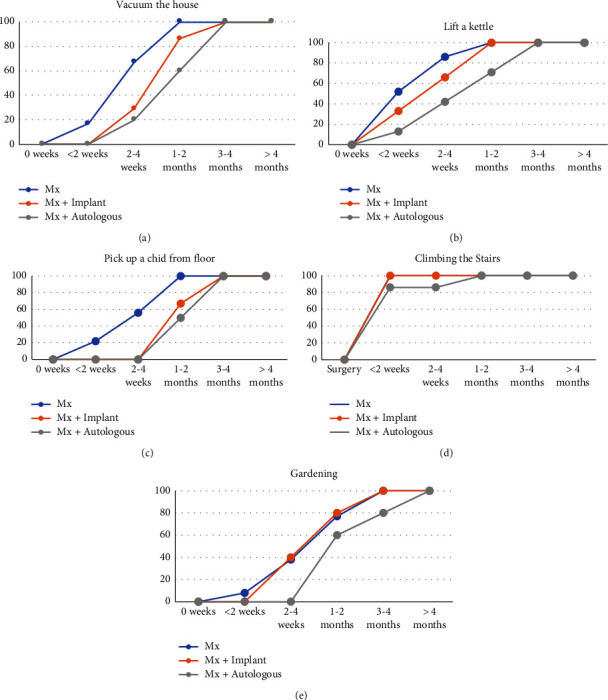
Time taken to return to activities of managing a household: (a) vacuuming the house (Mx = 18, Mx + implant = 7, and Mx + autologous = 5), (b) lifting the kettle (Mx = 21, Mx + implant = 6, and Mx + autologous = 7), (c) picking up a child from the floor (Mx = 7, Mx + implant = 3, and Mx + autologous = 3), (d) climbing the stairs (Mx and Mx + implant have the same results) (Mx = 16, Mx + implant = 6, and Mx + autologous = 7), and (e) gardening (Mx = 13, Mx + implant = 5, and Mx + autologous = 5), comparing simple mastectomy, implant-based reconstruction, and autologous reconstruction. Data are presented as the percentage of women who are able to perform the ADL at each time point. The number in brackets is the number of women who attempted this ADL during the recovery period.

Patients who underwent a simple mastectomy were able to complete ADLs associated with managing the household (including to vacuum the house, lift a kettle, pick up a child from floor, and gardening) earlier than patients undergoing a mastectomy and implant reconstruction or patients undergoing mastectomy and autologous reconstruction (Figure 4). Specifically, all patients who underwent a simple mastectomy could vacuum their house (*n* = 18, 4 did not attempt this activity), lift a kettle (*n* = 21, one did not attempt this activity), and pick a child off the floor (*n* = 9, 13 did not attempt this activity), where relevant, by 1-2 months. In comparison, 86% (*n* = 6), 100% (*n* = 6, 1 did not attempt this activity), and 67% (*n* = 2, 4 did not attempt this activity) of patients who underwent implant reconstruction could vacuum their house, lift a kettle, and pick a child off the floor by 1-2 months, and 60% (*n* = 3, 2 did not attempt this), 71% (*n* = 5), and 33% (*n* = 1, 4 did not attempt this) of patients undergoing an autologous reconstruction could vacuum their house, lift a kettle, and pick a child off the floor by 1-2 months. The majority, 97% (*n* = 32, 3 did not attempt this activity), of all patients could climb the stairs by 2 weeks. The majority, 77% (*n* = 10, 9 did not attempt) and 80% (*n* = 4, 2 did not attempt this activity), of patients who underwent simple mastectomy or implant-based reconstruction had returned to gardening within 1-2 months, compared with 60% (*n* = 3, 2 patients did not attempt this) of patients in the autologous group.

### 3.6. Fitness

Of the patients attempting sporting activities, 55% (*n* = 5, 13 did not attempt the activity) who underwent a simple mastectomy and 66% (*n* = 2, 4 did not attempt the activity) who underwent implant-based reconstruction could do so by 1-2 months, whereas it took 3-4 months for 50% (*n* = 2, 3 did not attempt the activity) of women who underwent autologous reconstruction to return to these activities (Supplementary Figure 1).

### 3.7. Work

In the study, 51% (*n* = 22) of patients were employed; of those that stated, seven were desk workers, five manual workers, and eight classed themselves as a mix of the two. Of the 22 employed patients, 73% (*n* = 16) returned to work. After simple mastectomy, 75% (*n* = 3) returned to work by 3-4 months, whereas 80% (*n* = 4) had returned to work by 1-2 months after implant-based reconstruction. After autologous reconstruction, 100% (*n* = 5) had returned to work by 3-4 months (Supplementary Figure 2).

### 3.8. Factors Contributing to Delay in Return to ADLs

Factors that delayed their return to ADLs were noted by 15 participants. Reasons included feeling underprepared for the procedure and unprepared for postoperative recovery with regard to the impact of drains, pain, and dressings. Pain was the most common factor which patients perceived to contribute to a delay in their return to ADLs (19%), followed by psychological impact (12%) and dressings (12%). Dressings specifically were commented alongside drains, suggesting the drain dressings were a particular problem.

## 4. Discussion

Currently, there are limited data available in the medical literature about time recommendations for return to ADLs after breast surgery. This has previously resulted in doctors recommending unrealistic times for return to work postoperatively [6, 8]. It is important we give patients accurate information to aid in the decision-making process. Our study found that 73% of patients returned to work, at a median of three to four months. There is significant variation in the literature with a meta-analysis demonstrating return to work after breast surgery ranging from 5.6% to 56.3% [9]. Other studies demonstrated a 60% return to work rate at 24 months after breast cancer surgery and an 80% return to work rate after a median time of 11.5 months [4, 8]. Factors associated with higher return to work rates included higher household income, breast conserving surgery, and adjustments to work duties, whereas factors associated with a reduced return to work rate included endocrine therapy and depression diagnosed after breast cancer diagnosis [4]. A return to work rate after breast surgery of 85% demonstrates that this type of surgery possibly has less effect on patients' future ability to work. The reason for a high return to work rate will be multifactorial, with some of the reasons stated above, however, could also be related to the extent of surgery and the difference in physical impact this may have in comparison to other surgeries or the overall success of treatment for breast cancer in comparison to other cancers. As other studies have demonstrated, we also found no difference in return to work time between different surgical procedures [8]. However, as there are differences in return to other ADLs between surgical procedures, return to work is not a sensitive nor specific measure of return to “normal” life.

Although not looking at specific ADLs, a study looking at return of physical activity levels after breast cancer surgery using the physical activity computerised questionnaire found that preoperative physical activity levels were not recovered at one year [10]. Based on the free text comments, pain was most likely the main factor relating to delays in return to activities. However, a large study demonstrated lower pain scores and narcotic use in autologous reconstruction than implant-based reconstruction [11]. Within implant-based reconstruction, there are possible differences between techniques, e.g., prepectoral vs. subpectoral reconstruction [12]. Five patients, two of whom had a simple mastectomy, commented specifically on difficulties with the drain. Drain-free mastectomy would alleviate this problem; however, it would need to be balanced against the potential increased need for drainage of seroma postoperatively [13, 14].

Another factor attributable to delay in returning to ADLs was chemotherapy. This study did not account for adjuvant therapy. Both chemotherapy and radiotherapy are associated with fatigue and other side effects [15, 16] which could significantly impact return to ADLs. This feasibility study demonstrates that recording of ADLs is feasible and does allow differences to be demonstrated between types of breast procedures. Allowing participants to fill in a questionnaire over a long time period (up to four months) after initial surgery potentially led to a poor return rate. Contacting the participants at set time points during the study period may encourage complete responses. There were a number of patients who did not fill in which type of surgery they underwent, which reduced the numbers for subgroup analysis. The option of “not attempted activity” was a major pitfall to the analysis and interpretation of results as we were unable to ascertain the reason why it was not attempted, e.g. because they could not perform the activity within the time frame or this was not an activity they would usually perform. A baseline assessment of the activities would be performed in the follow-up study. Certain questions such as lifting a child were also of little value as they were aimed at a very small subset of patients. Other important factors which may impact return to ADLs such as adjuvant therapy and delayed reconstruction were not included. Patient social demographics such as living alone or being the breadwinner may also impact return to ADLs and would be useful information to collect in the follow-up study. The use of electronic resources as reminders to complete the questionnaire or as a survey prompt at each of the allotted time points may increase response rate and accuracy. Including oncoplastic breast conservation surgery in the study such as mammoplasty surgery and local perforator flaps would also be beneficial.

## 5. Conclusion

The study proves the methodology to be feasible; however, it would benefit from extra data points and electronic questionnaires with reminders to improve recruitment. Despite being a small study, it does differentiate between the different reconstructive procedures and demonstrate differences in the speed of return to ADLs. This information can be incorporated into our preoperative patient information. This will enable a more thorough and informative consent process. The follow-up study due to start recruitment at the end of 2023 will strengthen these findings.

## Figures and Tables

**Table 1:** List of activities of daily living used in the questionnaire.

Theme	Activity
Personal hygiene	Getting in bath unassisted
	Getting out of bath unassisted
	Brushing your hair

Transportation and shopping	Socialise outside the house
	Driving
	Doing your own shopping

Managing household	Vacuum the house
	Lifting a kettle
	Pick up child from floor
	Climb the stairs
	Gardening

Fitness	Perform the postoperative exercises comfortably
	Return to playing sports/gym
	Wear a usual bra

Work	Return to work

## Data Availability

The data from this study, used to compile the tables and figures, are available in the supplementary information file. For any further information, contact the corresponding author.

## Supplementary Materials

Supplementary Materials Supplementary Table 1: questionnaire. Supplementary Table 2: frequency (%) of time to return to activities. Supplementary Figure 1: time taken to return to activities of fitness—(a) perform postoperative exercise comfortably (Mx = 21, Mx + implant = 7, and Mx + autologous = 7), (b) play sports or gym (Mx = 9, Mx + implant = 3, and Mx + autologous = 4), and (c) wear a usual bra (Mx = 13, Mx + implant = 3, and Mx + autologous = 5), comparing simple mastectomy, implant-based reconstruction, and autologous reconstruction. Data are presented as the percentage of women who are able to perform the ADL at each time point. The number in brackets is the number of women who attempted this ADL during the recovery period. Supplementary Figure 2: time taken to return to work (Mx = 4, Mx + implant = 5, and Mx + autologous = 5) comparing simple mastectomy, implant-based reconstruction, and autologous reconstruction. Data are presented as the percentage of women who are able to perform the ADL at each time point. The number in brackets is the number of women who attempted this ADL during the recovery period.
