# Supplementary material for: Return to Activities of Daily Living after Breast Cancer Surgery: An Observational Prospective Questionnaire-Based Study of Patients Undergoing Mastectomy with or without Immediate Reconstruction
Source: Breast J. 2023 Sep 20;2023:9345780. doi: 10.1155/2023/9345780 (PMC10533274; doi:10.1155/2023/9345780)
Supplement: Supplementary Materials — Supplementary Table 1: questionnaire. Supplementary Table 2: frequency (%) of time to return to activities. Supplementary Figure 1: time taken to return to activities of fitness—(a) perform postoperative exercise comfortably (Mx = 21, Mx + implant = 7, and Mx + autologous = 7), (b) play sports or gym (Mx = 9, Mx + implant = 3, and Mx + autologous = 4), and (c) wear a usual bra (Mx = 13, Mx + implant = 3, and Mx + autologous = 5), comparing simple mastectomy, implant-based reconstruction, and autologous reconstruction. Data are presented as the percentage of women who are able to perform the ADL at each time point. The number in brackets is the number of women who attempted this ADL during the recovery period. Supplementary Figure 2: time taken to return to work (Mx = 4, Mx + implant = 5, and Mx + autologous = 5) comparing simple mastectomy, implant-based reconstruction, and autologous reconstruction. Data are presented as the percentage of women who are able to perform the ADL at each time point. The number in brackets is the number of women who attempted this ADL during the recovery period. [file 9345780.f1.docx]

**Supplementary Table 1.** Questionnaire

Age ___________ Surgery____________________________________

Type of work (please circle) Manual Desk Mixed Retired

Please tick when you could:

| **Activity** | **<2**  **week** | **2-4**  **week** | **1-2**  **Month** | **3-4**  **month** | **>4 month** | **Not attempted** | |
| --- | --- | --- | --- | --- | --- | --- | --- |
| Get in the bath  (unassisted) |  |  |  |  |  |  |  |
| Get out of the bath (unassisted) |  |  |  |  |  |  |  |
| Brush your hair |  |  |  |  |  |  |  |
| Lift a full kettle |  |  |  |  |  |  |  |
| Climb the stairs  (unassisted) |  |  |  |  |  |  |  |
| Pick up a child from the floor |  |  |  |  |  |  |  |
| Vacuum the house |  |  |  |  |  |  |  |
| Gardening |  |  |  |  |  |  |  |
| Socialise outside of the house |  |  |  |  |  |  |  |
| Do your own shopping |  |  |  |  |  |  |  |
| Drive |  |  |  |  |  |  |  |
| Return to work |  |  |  |  |  |  |  |
| Perform post-op exercises comfortably |  |  |  |  |  |  |  |
| Wear usual bras |  |  |  |  |  |  |  |
| Go back to the gym / playing sports |  |  |  |  |  |  |  |

Is there anything which you felt delayed your return to any of the above activities? (Please circle)

Dressing issues Pain Psychologically not ready

Following your operation, is there any further information which you were not aware of which you would have liked to have been told about prior to your surgery?

………………………………………………………..………………………………………………………..………………………………………………………..………………………………………………………..………………………………………………………..…………………………………………………………………………………………………………….

**Supplementary Table 2. Frequency (%) of time to return to activities**

|  |  | <2weeks | 2-4 weeks | 1-2 months | 3-4 months | > 4 months | Did not attempt |
| --- | --- | --- | --- | --- | --- | --- | --- |
| **Getting in bath unassisted** | **Mastectomy (n=22)** | 14 | 2 | 0 | 0 | 0 | 6 |
|  |  | 64% | 9% | 0% | 0% | 0% | 27% |
|  | **Implant (n=7)** | 3 | 1 | 1 | 1 | 0 | 1 |
|  |  | 43% | 14% | 14% | 14% | 0% | 14% |
|  | **Autologous (n=7)** | 3 | 1 | 2 | 1 | 0 | 0 |
|  |  | 43% | 14% | 29% | 14% | 0% | 0% |
|  | **Total (n=36)** | 20 | 4 | 3 | 2 | 0 | 7 |
|  |  | 56% | 11% | 8% | 6% | 0% | 19% |
|  | **Operation**  **not stated (n= 6)** | 5 | 1 | 0 | 0 | 0 | 0 |
|  |  | 83% | 17% | 0% | 0% | 0% | 0% |
|  | **All in. operation not stated ( n = 42)** | 25 | 5 | 3 | 2 | 0 | 7 |
|  |  | 60% | 12% | 7% | 5% | 0% | 17% |
| **Getting out bath unassisted** | **Mastectomy (n=22)** | 14 | 2 | 0 | 0 | 0 | 6 |
|  |  | 64% | 9% | 0% | 0% | 0% | 27% |
|  | **Implant (n=7)** | 3 | 2 | 0 | 1 | 0 | 1 |
|  |  | 43% | 29% | 0% | 14% | 0% | 14% |
|  | **Autologous (n=7)** | 3 | 1 | 1 | 2 | 0 | 0 |
|  |  | 43% | 14% | 14% | 29% | 0% | 0% |
|  | **All (n=36)** | 20 | 5 | 1 | 3 | 0 | 7 |
|  |  | 56% | 14% | 3% | 8% | 0% | 19% |
|  | **Operation not stated (n= 6)** | 4  67% | 0  0% | 2  33% | 0  0% | 0  0% | 0  0% |
|  | **All inc. operation not stated (n=42)** | 24 | 5 | 3 | 3 | 0 | 7 |
|  |  | 57% | 12% | 7% | 7% | 0% | 17% |
| **Brush your hair** | **Mastectomy (n=22)** | 18 | 1 | 1 | 0 | 0 | 2 |
|  |  | 82% | 5% | 5% | 0% | 0% | 50% |
|  | **Implant (n=7)** | 5 | 1 | 0 | 0 | 0 | 1 |
|  |  | 71% | 14% | 0% | 0% | 0% | 50% |
|  | **Autologous (n=7)** | 6 | 0 | 1 | 0 | 0 | 0 |
|  |  | 86% | 0% | 14% | 0% | 0% | 0% |
|  | **All (n=36)** | 29 | 2 | 2 | 0 | 0 | 3 |
|  |  | 81% | 6% | 6% | 0% | 0% | 7% |
|  | **Operation not stated (n=6)** | 6 | 0 | 0 | 0 | 0 | 0 |
|  |  | 100% | 0% | 0% | 0% | 0% | 0% |
|  | **All inc. operation not stated (n=42)** | 35 | 2 | 2 | 0 | 0 | 3 |
|  |  | 83% | 5% | 5% | 0% | 0% | 7% |
|  |  |  |  |  |  |  |  |
|  |  |  |  |  |  |  |  |
|  |  |  |  |  |  |  |  |
|  |  |  |  |  |  |  |  |
|  |  | <2weeks | 2-4 weeks | 1-2 months | 3-4 months | > 4 months | Did not attempt |
| **Lift a kettle** | **Mastectomy (n=22)** | 11 | 7 | 3 | 0 | 0 | 1 |
|  |  | 50% | 32% | 14% | 0% | 0% | 5% |
|  | **Implant (n=7)** | 2 | 2 | 2 | 0 | 0 | 1 |
|  |  | 29% | 29% | 29% | 0% | 0% | 14% |
|  | **Autologous (n=7)** | 1 | 2 | 2 | 2 | 0 | 0 |
|  |  | 14% | 29% | 29% | 29% | 0% | 0% |
|  | **All (n=36)** | 14 | 11 | 7 | 2 | 0 | 2 |
|  |  | 39% | 31% | 19% | 6% | 0% | 6% |
|  | **Operation not stated (n=6)** | 2 | 3 | 0 | 1 | 0 | 0 |
|  |  | 33% | 50% | 0% | 17% | 0% | 0% |
|  | **All inc. operation not stated (n=42)** | 16 | 14 | 7 | 3 | 0 | 2 |
|  |  | 38% | 33% | 17% | 7% | 0% | 5% |
| **Climb the stairs** | **Mastectomy (n=22)** | 20 | 0 | 0 | 0 | 0 | 2 |
|  |  | 91% | 0% | 0% | 0% | 0% | 9% |
|  | **Implant (n=7)** | 6 | 0 | 0 | 0 | 0 | 1 |
|  |  | 86% | 0% | 0% | 0% | 0% | 14% |
|  | **Autologous (n=7)** | 6 | 0 | 1 | 0 | 0 | 0 |
|  |  | 86% | 0% | 14% | 0% | 0% | 0% |
|  | **All (n=36)** | 32 | 0 | 1 | 0 | 0 | 3 |
|  |  | 89% | 0% | 3% | 0% | 0% | 8% |
|  | **Operation not stated (n=6)** | 6 | 0 | 0 | 0 | 0 |  |
|  |  | 100% | 0% | 0% | 0% | 0% | 0% |
|  | **All inc. operation not stated ( n=42)** | 38 | 0 | 1 | 0 | 0 | 3 |
|  |  | 90% | 0% | 2% | 0% | 0% | 7% |
| **Pick up child from floor** | **Mastectomy (n=22)** | 1 | 3 | 3 | 0 | 0 | 15 |
|  |  | 5% | 14% | 14% | 0% | 0% | 68% |
|  | **Implant (n=7)** | 0 | 0 | 2 | 1 | 0 | 4 |
|  |  | 0% | 0% | 29% | 14% | 0% | 57% |
|  | **Autologous (n=7)** | 0 | 0 | 1 | 2 | 0 | 4 |
|  |  | 0% | 0% | 14% | 29% | 0% | 57% |
|  | **All (n=36)** | 1 | 3 | 6 | 3 | 0 | 23 |
|  |  | 3% | 8% | 17% | 8% | 0% | 64% |
|  | **Operation not stated (n=6)** | 0 | 2 | 0 | 0 | 0 | 4 |
|  |  | 0% | 33% | 0% | 0% | 0% | 67% |
|  | **All inc. operation not stated ( n=42)** | 1 | 5 | 6 | 3 | 0 | 27 |
|  |  | 2% | 12% | 14% | 7% | 0% | 64% |
|  |  |  |  |  |  |  |  |
|  |  | <2weeks | 2-4 weeks | 1-2 months | 3-4 months | > 4 months | Did not attempt |
| **Vacuum the house** | **Mastectomy (n=22)** | 3 | 9 | 6 | 0 | 0 | 4 |
|  |  | 14% | 41% | 27% | 0% | 0% | 18% |
|  | **Implant (n=7)** | 0 | 2 | 4 | 1 | 0 | 0 |
|  |  | 0% | 29% | 57% | 14% | 0% | 0% |
|  | **Autologous (n=7)** | 0 | 1 | 2 | 2 | 0 | 2 |
|  |  | 0% | 14% | 29% | 29% | 0% | 29% |
|  | **All (n=36)** | 3 | 12 | 12 | 3 | 0 | 6 |
|  |  | 8% | 33% | 33% | 8% | 0% | 17% |
|  | **Operation not stated (n=6)** | 1 | 2 | 1 | 0 | 0 | 2 |
|  |  | 17% | 33% | 17% | 0% | 0% | 33% |
|  | **All inc. operation not stated (n = 42)** | 4 | 14 | 13 | 3 | 0 | 8 |
|  |  | 10% | 33% | 31% | 7% | 0% | 19% |
| **Gardening** | **Mastectomy (n=22)** | 1 | 4 | 5 | 3 | 0 | 9 |
|  |  | 5% | 18% | 23% | 14% | 0% | 41% |
|  | **Implant n=7** | 0 | 2 | 2 | 1 | 0 | 2 |
|  |  | 0% | 29% | 29% | 14% | 0% | 29% |
|  | **Autologous (n=7)** | 0 | 0 | 3 | 1 | 1 | 2 |
|  |  | 0% | 0% | 43% | 14% | 14% | 29% |
|  | **All (n=36 )** | 1 | 6 | 10 | 5 | 1 | 13 |
|  |  | 3% | 17% | 28% | 14% | 3% | 36% |
|  | **Operation not stated (n=6)** | 1 | 1 | 0 | 0 | 0 | 4 |
|  |  | 17% | 17% | 0% | 0% | 0% | 67% |
|  | **All inc. operation not stated (n = 42 )** | 2 | 7 | 10 | 5 | 1 | 17 |
|  |  | 5% | 17% | 24% | 12% | 2% | 40% |
| **Socialise outside the house** | **Mastectomy (n=22)** | 11 | 8 | 2 | 0 | 0 | 1 |
|  |  | 50% | 36% | 9% | 0% | 0% | 5% |
|  | **Implant (n=7)** | 3 | 3 | 0 | 0 | 0 | 1 |
|  |  | 43% | 43% | 0% | 0% | 0% | 14% |
|  | **Autologous (n=7)** | 1 | 3 | 2 | 1 | 0 | 0 |
|  |  | 14% | 43% | 29% | 14% | 0% | 0% |
|  | **All (n=36)** | 15 | 14 | 4 | 1 | 0 | 2 |
|  |  | 42% | 39% | 11% | 3% | 0% | 6% |
|  | **Operation not stated (n=6)** | 2 | 3 | 1 | 0 | 0 | 0 |
|  |  | 33% | 50% | 17% | 0% | 0% | 0% |
|  | **All inc. operation not stated (n = 42)** | 17 | 17 | 5 | 1 | 0 | 2 |
|  |  | 40% | 40% | 12% | 2% | 0% | 5% |
|  |  |  |  |  |  |  |  |
|  |  | <2weeks | 2-4 weeks | 1-2 months | 3-4 months | > 4 months | Did not attempt |
| **Do your own shopping** | **Mastectomy (n=22)** | 4 | 9 | 5 | 0 | 0 | 4 |
|  |  | 18% | 41% | 23% | 0% | 0% | 18% |
|  | **Implant (n=7)** | 2 | 3 | 1 | 0 | 0 | 1 |
|  |  | 29% | 43% | 14% | 0% | 0% | 14% |
|  | **Autologous (n=7)** | 0 | 2 | 2 | 3 | 0 | 0 |
|  |  | 0% | 29% | 29% | 43% | 0% | 0% |
|  | **All (n=36)** | 6 | 14 | 8 | 3 | 0 | 5 |
|  |  | 17% | 39% | 22% | 8% | 0% | 14% |
|  | **Operation not stated (n=6)** | 2 | 2 | 2 | 0 | 0 | 0 |
|  |  | 33% | 33% | 33% | 0% | 0% | 0% |
|  | **All inc. operation not stated (n = 42)** | 8 | 16 | 10 | 3 | 0 | 5 |
|  |  | 19% | 38% | 24% | 7% | 0% | 12% |
| **Drive** | **Mastectomy (n=22)** | 2 | 8 | 4 | 0 | 0 | 8 |
|  |  | 9% | 36% | 18% | 0% | 0% | 36% |
|  | **Implant (n=7)** | 1 | 4 | 1 | 0 | 0 | 1 |
|  |  | 14% | 57% | 14% | 0% | 0% | 14% |
|  | **Autologous (n=7)** | 0 | 2 | 1 | 3 | 0 | 1 |
|  |  | 0% | 29% | 14% | 43% | 0% | 14% |
|  | **All (n=36)** | 3 | 14 | 6 | 3 | 0 | 10 |
|  |  | 8% | 39% | 17% | 8% | 0% | 28% |
|  | **Operation not stated (n=6)** | 0 | 2 | 0 | 1 | 0 | 3 |
|  |  | 0% | 33% | 0% | 17% | 0% | 50% |
|  | **All inc. operation not stated (n = 42)** | 3 | 16 | 6 | 4 | 0 | 13 |
|  |  | 7% | 38% | 14% | 10% | 0% | 31% |
| **Return to work** | **Mastectomy (n=22)** | 0 | 0 | 1 | 2 | 1 | 18 |
|  |  | 0% | 0% | 5% | 9% | 5% | 82% |
|  | **Implant (n=7)** | 1 | 0 | 3 | 1 | 0 | 2 |
|  |  | 14% | 0% | 43% | 14% | 0% | 29% |
|  | **Autologous (n=7)** | 0 | 0 | 1 | 4 | 0 | 2 |
|  |  | 0% | 0% | 14% | 57% | 0% | 29% |
|  | **All (n=36)** | 1 | 0 | 5 | 7 | 1 | 22 |
|  |  | 3% | 0% | 14% | 19% | 3% | 61% |
|  | **Operation not stated (n=6)** | 0 | 2 | 0 | 0 | 0 | 4 |
|  |  | 0% | 33% | 0% | 0% | 0% | 67% |
|  | **All inc. operation not stated ( n = 42)** | 1 | 2 | 5 | 7 | 1 | 26 |
|  |  | 2% | 5% | 12% | 17% | 2% | 62% |
|  |  |  |  |  |  |  |  |
|  |  | <2weeks | 2-4 weeks | 1-2 months | 3-4 months | > 4 months | Did not attempt |
| **Perform exercises comfortably** | **Mastectomy (n=22)** | 8 | 9 | 2 | 2 | 0 | 1 |
|  |  | 36% | 41% | 9% | 9% | 0% | 5% |
|  | **Implant (n=7)** | 5 | 1 | 1 | 0 | 0 | 0 |
|  |  | 71% | 14% | 14% | 0% | 0% | 0% |
|  | **Autologous (n=7)** | 3 | 1 | 2 | 0 | 1 | 0 |
|  |  | 43% | 14% | 29% | 0% | 14% | 0% |
|  | **All (n=36)** | 16 | 11 | 5 | 2 | 1 | 1 |
|  |  | 44% | 31% | 14% | 6% | 3% | 3% |
|  | **Operation not stated (n=6)** | 4 | 1 | 1 |  |  |  |
|  |  | 67% | 17% | 17% | 0% | 0% | 0% |
|  | **All inc. operation not stated (n=42)** | 20 | 12 | 6 | 2 | 1 | 1 |
|  |  | 48% | 29% | 14% | 5% | 2% | 2% |
| **Wear usual bra** | **Mastectomy (n=22)** | 4 | 3 | 4 | 1 | 1 | 9 |
|  |  | 18% | 14% | 18% | 5% | 5% | 41% |
|  | **Implant (n=7)** | 0 | 0 | 2 | 0 | 1 | 4 |
|  |  | 0% | 0% | 29% | 0% | 14% | 57% |
|  | **Autologous (n=7)** | 2 | 0 | 3 | 1 | 0 | 1 |
|  |  | 29% | 0% | 43% | 14% | 0% | 14% |
|  | **All (n=36)** | 6 | 3 | 9 | 2 | 2 | 14 |
|  |  | 17% | 8% | 25% | 6% | 6% | 39% |
|  | **Operation not stated (n=6)** | 1 | 1 | 2 | 0 | 0 | 2 |
|  |  | 17% | 17% | 33% | 0% | 0% | 33% |
|  | **All inc. operation not stated (n=42)** | 7 | 4 | 11 | 2 | 2 | 16 |
|  |  | 17% | 10% | 26% | 5% | 5% | 38% |
| **Play sport** | **Mastectomy (n=22)** | 0 | 3 | 5 | 0 | 1 | 13 |
|  |  | 0% | 14% | 23% | 0% | 5% | 59% |
|  | **Implant (n=7)** | 0 | 0 | 2 | 1 | 0 | 4 |
|  |  | 0% | 0% | 29% | 14% | 0% | 57% |
|  | **Autologous (n=7)** | 1 | 0 | 0 | 2 | 1 | 3 |
|  |  | 14% | 0% | 0% | 29% | 14% | 43% |
|  | **All (n=36)** | 1 | 3 | 7 | 3 | 2 | 20 |
|  |  | 3% | 8% | 19% | 8% | 6% | 56% |
|  | **Operation not stated (n=6)** | 0 | 0 | 2 | 0 | 0 | 4 |
|  |  | 0% | 0% | 33% | 0% | 0% | 67% |
|  | **All inc. operation not stated ( n = 42)** | 1 | 3 | 9 | 3 | 2 | 24 |
|  |  | 2% | 7% | 21% | 7% | 5% | 57% |

a. b.

c.

**Supplementary Figure 1.** Time taken to return to activities of fitness a. perform post-operative exercise comfortably (Mx=21, Mx + implant=7, Mx + autologous=7), b. play sport or gym (Mx=9, Mx + implant=3, Mx + autologous=4) and c. wear a usual bra (Mx=13, Mx + implant=3, Mx + autologous=5) comparing simple mastectomy, implant based reconstruction and autologous reconstruction. Data presented as the percentage of women who are able to performing the ADL at each time point. The number in brackets is the number of women who attempted this ADL during the recovery period

**Supplementary Figure 2.** Time taken to return to work (Mx=4, Mx + implant=5, Mx + autologous=5) comparing simple mastectomy, implant based reconstruction and autologous reconstruction. Data presented as the percentage of women who are able to performing the ADL at each time point. The number in brackets is the number of women who attempted this ADL during the recovery period
